# The Artificial Feeding of Infants

**Published:** 1902-09

**Authors:** 


The Artificial Feeding of Infants. By Charles F. Judson, M. D., Physician to the Medical Dispensary of the Children's Hospital, and J. Claxton Gittings, M. D., Assistant Physician to the Medical Dispensary of the Children's Hospital. Duodecimo, pp. 368. Philadelphia: J. B. Lippincott Company. 1902. [Price, $2.00.]

Judson and Gittings present in this volume an authoritative statement of the views of leading pediatrists in Europe and

America upon the subject of artificial feeding at the present day. They state in the preface that the substance of the work has been gleaned from the periodical literature, monographs and textbooks published since 1894, and the extensive bibliography at the end of the book shows the range and value of their gleaning.

The subject of breastfeeding is not considered at all nor any of the related problems, as the improvement of the mother's milk.

The book contains several practical chapters on the subject of infant feeding. It serves also to acquaint the reader with the views of the prominent pediatrists of the day on all matters relating to artificial feeding, and presents to him in concise form information which would otherwise take much time and labor to acquire. M. J. F.

